# The Effect of Water- and Ultrasonic Bath Systems on Bioactive Compounds and Fatty Acid Compositions of Unroasted and Roasted Pumpkin Seeds

**DOI:** 10.3390/foods14152740

**Published:** 2025-08-05

**Authors:** Isam A. Mohamed Ahmed, Mehmet Musa Özcan, Nurhan Uslu, Emad Karrar, Fahad Aljuhaimi

**Affiliations:** 1 Department of Food Science & Nutrition, College of Food and Agricultural Sciences, King Saud University, Riyadh 11451, Saudi Arabia; iali@ksu.edu.sa (I.A.M.A.); faljuhaimi@ksu.edu.sa (F.A.); 2 Department of Food Engineering, Faculty of Agriculture, Selcuk University, Konya 42031, Turkey; nurhanuslu.gmuh@gmail.com; 3 Department of Plant Sciences, North Dakota State University, Fargo, ND 58108, USA; emad.karrar@ndsu.edu

**Keywords:** pumpkin seeds, roasting, extraction, antioxidant activity, fatty acids, phenolics

## Abstract

In this study, the effects of water bath and ultrasonic bath systems on bioactive properties, phenolic components and fatty acid profiles of unroasted and roasted pumpkin seeds were investigated. It is thought that determining the bioactive components, phenolic constituents and fatty acid profiles of unroasted and roasted pumpkin seeds will lead to the establishment of usage norms according to their composition characteristics. Total phenolic quantities of the pumpkin seed extracts obtained by water bath extraction of the seeds were defined to be between 7.58 (control) and 11.55 (25 min) and 10.20 (control) and 17.18 mg GAE/100 g (50 min), respectively. Phenolic content increased by 50% after 50 min of ultrasonic extraction, indicating the efficiency of this method. Also, total flavonoid amounts increased about 55% after 25 min of ultrasonic extraction, indicating the efficiency of this method. It was observed that the catechin contents of unroasted pumpkin seeds obtained in water and ultrasonic baths decreased significantly at the 50th minute of extraction compared to the control. The antioxidant activity values (DPPH) of roasted pumpkin seeds treated in water- and ultrasonic bath systems increased by approximately 10% compared to the control at 50 min of sonication in both systems, respectively. Also, the 3,4-dihydroxybenzoic acid amounts of the extracts obtained by both extraction systems of roasted pumpkin seeds were determined between 9.85 (50 min) and 17.22 mg/100 g (control) and 11.17 (25 min) and 13.74 mg/100 g (50 min), respectively. The linoleic acid amounts of unroasted pumpkin seed oils extracted in water- and ultrasonic baths varied between 52.34 (50 min) and 53.33% (control) to 52.90 (50 min) and 53.04% (control), respectively. The linoleic acid values of the roasted pumpkin seed oils were established to be between 52.30 (50 min) and 52.84 (25 min) and 52.32 (50 min) and 53.46% (25 min), respectively. In general, the phenolic compound amounts of roasted pumpkin seeds were higher than those of unroasted ones. The fatty acid amounts of pumpkin seed oils extracted with an ultrasonic bath were generally slightly higher than those extracted with a water bath. In future studies, changes in the phytochemical and bioactive properties of pumpkin seed oils obtained by applying different roasting techniques and extraction methods will be investigated.

## 1. Introduction

Most of the pumpkin seeds have been considered waste from the past to the present [1]. It has been reported that pumpkin is rich in phytochemicals [1,2]. The pumpkin plant species whose seeds are used to obtain vegetable oil in Turkey is mostly *Cucurbita pepo* L. Thanks to the climate and soil conditions of our country, it is possible to grow other pumpkin plant species without any problems. Pumpkin seeds are preferred to be consumed as a snack rather than as a source of oil in Mediterranean and Middle Eastern countries [3]. The inner part of the pumpkin seed, which has many benefits and areas of use, contains around 45–50% polyunsaturated fatty acids and a dark green fixed oil rich in antioxidants. Pumpkin seed oil, which is very rich in vitamin E (especially γ-tocopherol), contains high levels of vitamins A and B, phytosterols and the amino acid called cucurbitin [3,4]. Depending on the type of seed in these species belonging to the cucurbitaceae family, it contains 30–50% oil; 1% steroid; 25–55% protein; 6–10% carbohydrate; and cucurbitacin agent, tocols, trace elements, vitamins and other glycosides [4]. In addition to food consumption, pumpkin seeds, which are also used for medical and cosmetic purposes, are a rich source of nutrients in terms of the minerals, vitamins, proteins and oils they contain [3]. Pumpkin fruit is among the daily meals, and it is the basic ingredient of foods such as soups, desserts, salads, pickling and hash browns [3,4]. Cucurbita pulp and seed are used for many purposes in the food industry. Pumpkin seeds are both delicious and nutritious snacks on our tables, and traditionally provide energy and health support, and provide many benefits to our bodies with their high nutritional values [4]. Pumpkin seed oil, which has rich functional properties, is used in many countries as cooking oil, in salads, and in bread and pastry making by obtaining it through the cold press method. In addition, pumpkin seed oil is very rich in vitamins and minerals [5]. It contains omega-3, omega-6, vitamins A (retinol) and E (tocopherol), fiber, iron, zinc and selenium minerals [4,5]. This oil, which is not preferred in meals due to its color and smell, is preferred as salad oil in some countries due to its important bioactive components. The main reason why oil ratios and other specified bioactive component contents vary according to pumpkin plant species is stated as climate changes and genetic factors of the region where the plant is grown [3,4,5]. It can be used as a raw material in many foods such as cookies, soup and bread. In addition to being used as a raw material in foods such as bread, grain products, salads and cakes, pumpkin is also consumed fresh. Pumpkin oil is also recommended for use in foods as an edible oil [6,7,8]. Cucurbita fruit is widely used in the health sector due to its nutritional properties, phytochemicals, medicinal use and traditionally good for many diseases [9,10]. Pumpkin seed oil has been found to contain significant amounts of unsaturated fatty acids (78.0%) and is a rich source of linoleic (47.0%), palmitic (13.3%), stearic (8.0%) and oleic (29.0%) [6]. In roasted pumpkin seeds, quinine was routinely reported at 123.39 µg/100 g, caffeic acid at 88.60 µg/100 g, quercetin at 35.09 µg/100 g and 1.92 µg/100 g in decreasing order [10]. Its seed is a rich nutritional source and its health benefits are well documented [11,12]. The roasting process causes non-enzymatic reactions that contribute to the taste, flavor, aroma and color of oilseeds such as hazelnuts and raw foods [13,14,15]. Ultrasound application in the extraction process is an alternative method that has an effect by mechanically breaking the cell walls and providing material transfer, and is faster than other extraction methods [16,17,18,19]. Therefore, it is thought that determining the bioactive components, phenolic constituents, and fatty acid profiles of unroasted and roasted pumpkin seeds will lead to the establishment of usage norms according to their composition characteristics. The aim of this study was to reveal the changes in total phenol, flavonoid contents, antioxidant activity values, phenolic compounds and fatty acid profiles of pumpkin seeds extracted after water bath and ultrasonic bath treatments applied to unroasted and roasted pumpkin seeds.

## 2. Materials and Methods

### 2.1. Materials

Pumpkin seeds were provided from Konya (Selçuklu, Turkey) province in Turkey in 2024. The seed coat was manually removed before roasting. The analyses were performed only on the cotyledon. Pumpkin seeds were dried in the open air for 1 month and stored closed in colored glass jars at +4 °C until analysis.

### 2.2. Methods

#### 2.2.1. Roasting Process

The cotyledon was used in the analyses performed in the study after being separated from the hard outer shell of the seed. The shell was removed as waste. The shelled seeds were laid out on a tray at a thickness of 2–3 mm and subjected to the roasting process. Roasting was carried out in a conventional oven (NUVE FN 055, Ankara, Turkey) at 160 °C for 23 min.

#### 2.2.2. Moisture Content

After the pumpkin seeds were shelled, moisture analysis was performed on the remaining cotyledons. The pumpkin seeds were cracked using forceps, and the shells were manually removed from the cotyledons. The moisture contents of pumpkin seed samples were characterized by the KERN & SOHN GmbH (Balingen, Germany) infrared moisture analyser.

#### 2.2.3. Color Values

L*, a* and b* values of pumpkin seeds were defined using a Minolta Chroma meter CR 400 according to the CIELab color scale [20].

#### 2.2.4. Oil Content

An amount of 2 g of ground seed sample was placed in a cartridge and kept in a bottle with 100 mL of petroleum ether (Merck, Darmstadt, Germany), and then they were shaken in a water bath (Wise Bath) and an ultrasonic bath (Bandelin Sonorex) for 25 and 50 min, respectively. Then, the oil samples obtained were used for fatty acid analyses.

#### 2.2.5. Extraction Procedure

The extraction of seeds was determined according to the method applied by Jakopic et al. [21]. Ground samples (5 g) were added to 15 mL of methanol. The mixture was kept in a water bath and an ultrasonic water bath for 0 min (control), 25 min and 50 min, followed by centrifugation at 6000 rpm for 10 min, and then the supernatant was filtered through a 0.45 µm membrane filter. After pretreatments, the dried extracts were dissolved in 10 mL of methanol.

#### 2.2.6. Total Phenolic Amount

Total phenolic amounts of seed extracts were defined using the Folin–Ciocalteu (FC) method based on the report proposed by Yoo et al. [22]. FC (1 mL) and Na_2_CO_3_ (10 mL) were added to extract and mixed with a vortex. The deionised water was added until the final volume was 25 mL, and kept in the dark for 1 h. After preprocessing, the absorbance was measured at 750 nm.

#### 2.2.7. Total Flavonoid Content

Total flavonoid contents of pumpkin seeds were determined by the method suggested by Hogan et al. [23] by adding 0.3 mL of NaNO_2_, 0.3 mL of AlCl_3_ and 2 mL of NaOH to 1 mL of extract and making the sample ready for analysis. After some pretreatments, the absorbance was measured at 510 nm.

#### 2.2.8. Antioxidant Activity

##### DPPH Free Radical Scavenging Activity

The antioxidant activities of pumpkin seed extracts were measured using DPPH (1,1-diphenyl-2-picrylhydrazyl) according to the report suggested by Lee et al. [24]. The extract was mixed with 2 mL of a methanolic solution of DPPH.

##### ABTS Free Radical Scavenging Activity

Antioxidant activity of extracts was determined according to the study of Köseoğlu et al. [25] using 2,2′-azinobis-3-ethylbenzothiazoline-6-sulfonic acid (ABTS). After preprocessing, the absorbance values were obtained at 734 nm.

#### 2.2.9. Determination of Phenolic Compounds

The analysis and chromatographic separations of phenolic constituents of pumpkin seeds shaken in water- and ultrasonic bath systems were carried out by HPLC (Shimadzu, SCL-10A VP) equipped with a PDA detector and an Inertsil ODS-3 (5 µm; 4.6 × 250 mm) column. The elution programme was employed: 0–0.10 min 8% B; 0.10–2 min 10% B; 2–27 min 30% B; 27–37 min 56% B; 37–37.10 min 8% B; 37.10–45 min 8% B. The total running time per sample was 60 min.

#### 2.2.10. Fatty Acid Profiling

Fatty acid methyl esters of pumpkin seed oil esterified according to the method proposed by Multari et al. [26] were characterized by gas chromatography (Shimadzu GC-2010) equipped with a flame-ionization detector (FID) and capillary column (Tecnocroma TR-CN100, 60 m × 0.25 mm, film thickness: 0.20 µm). The total flow rate was 80 mL/min, and the split rate was also 1/40. The temperature of the injection block and detector was 260 °C. The mobile phase was nitrogen with a 1.51 mL/min flow rate. The total flow rate was 80 mL/min, and the split rate was also 1/40. Column temperature was programmed at 120 °C for 5 min and increased to 240 °C at 4 °C/min and held for 25 min at 240 °C.

### 2.3. Statistical Analysis

JMP version 9.0 was used for analysis of variance (ANOVA). The results are mean ± standard deviation (MSTAT C) of independent roasting and shaking systems.

## 3. Results and Discussion

### 3.1. Physico-Chemical Properties of Unroasted and Roasted Pumpkin Seeds

Moisture amounts and color values of pumpkin seeds extracted in water- and ultrasonic baths are defined in Table 1. Initial moisture amounts of unroasted and roasted pumpkin seeds were depicted as 8.05 and 2.56%, respectively. In addition, initial color values of unroasted and roasted seeds were defined as 66.62 and 63.38 (L*), −2.14 and 1.83 (a*), and 14.68 and 25.15 (b*) (Table 1). The color parameter results of roasted pumpkin seeds created significant differences compared to unroasted ones. The color parameter results of roasted pumpkin seeds produced significant differences compared to unroasted ones, which may be due to the formation of Maillard reaction products, caramelization and thermal degradation of pigments during roasting. The brightness value (L*) of the seeds decreased with roasting.

### 3.2. Bioactive Properties of Unroasted and Roasted Pumpkin Seeds Extracted in Water Bath and Ultrasonic Bath

Bioactive compounds and antioxidant activity values of unroasted and roasted pumpkin seeds extracted in a water bath and ultrasonic bath for different times (25 and 50 min) are presented in Table 2. Heat treatment and both extraction systems were effective on the bioactive properties of pumpkin seeds. Total phenol amounts of the seed extracts were defined to be between 7.58 (control) and 11.55 mg GAE/100 g (25 min) and 10.20 (control) and 17.18 mg GAE/100 g (50 min), respectively.

Total phenol and total flavonoid contents of unroasted pumpkin seeds treated in water- and ultrasonic bath systems increased by 52% and 48%, and 66% and 31%, respectively, at 25 min of sonication compared to the control. In addition, total phenol contents of roasted pumpkin seeds treated in water- and ultrasonic bath systems increased by 68% and 44% compared to the control at 50 min of sonication, respectively. The water bath and ultrasound treatments may have disrupted the cells containing the bioactive compounds in the pumpkin seeds, increasing their release [19]. It was observed that the catechin contents of unroasted pumpkin seeds obtained in water and ultrasonic baths decreased significantly at the 50th minute of extraction compared to the control. Total flavonoid amounts of the extracts extracted by the extraction of roasted pumpkin seeds in both extraction systems were assigned between 48.81 (control) and 112.14 mg/100 g (25 min) and 48.81 (50 min) and 58.81 mg/100 g (control), respectively. Antioxidant activities of the extracts obtained from both extraction systems of unroasted and roasted pumpkin seeds showed partial changes depending on the extraction type and duration. The antioxidant activity values (DPPH) of roasted pumpkin seeds treated in water- and ultrasonic bath systems increased by approximately 10% compared to the control at 50 min of sonication in both systems, respectively. The highest antioxidant activity was determined in both extraction systems of roasted pumpkin seeds. This is probably due to Maillard reaction products formed as a result of heat treatment with the Folin–Ciocalteu reagent, which increased the total phenol amount of the extract and therefore increased the antioxidant activities of roasted pumpkin seeds [15]. Total phenol and flavonoid quantities of pumpkin seeds increased with increasing sonication time in both extraction systems. Water bath and ultrasonic bath treatments applied to both roasted and unroasted pumpkin seeds increased the bioactive properties of the seeds compared to the control. This increase sometimes resulted in significant increases at 25 and 50 min. This may be due to the agitation speed and the degree of impact of the ultrasonic waves and roasting temperature on the seed cells [19]. The total phenol, the total flavonoid and the antioxidant capacities were 4.28 and 13.35 mgGAE/g, 3.24 and 5.99 mgQE/g, and 0.03 and 0.04 mgTEAC/100 g in pumpkin seed samples, respectively [27]. In a previous study on pumpkin seeds, the total phenolic amounts of “Topak” pumpkin seeds varied between 4.91 (control) and 5.55 mg GAE/100 g (oven), while the total phenolic contents of “Sivri” pumpkin seeds were determined between 3.76 (control) and 5.09 mg GAE/100 g (oven) [28]. The monitored changes may have been due to factors such as harvest time, climatic factors and applied heat treatment.

### 3.3. Phenolic Constituents of Unroasted and Roasted Pumpkin Seeds

Phenolic compounds of unroasted and roasted pumpkin seeds extracted at different times (25 and 50 min) were identified by HPLC, and their amounts are shown in Table 3. It was observed that extraction types and times were effective on the phenolic compounds of pumpkin seeds. Catechin, gallic acid, 3,4-dihydroxybenzoic acid, rutin and caffeic acid were the predominant phenolic compounds of unroasted and roasted pumpkin seeds. While the gallic acid amounts of unroasted pumpkin seeds extracted in a water bath at different times varied between 23.31 (50 min) and 28.89 mg/100 g (control), the gallic acid quantities of pumpkin seeds extracted in ultrasonic bath were characterized to be between 2.31 (control) and 23.59 mg/100 g (25 min). The catechin contents of pumpkin seeds extracted in both extraction systems were determined to be between 44.50 (50 min) and 76.25 mg/100 g (25 min) and 48.59 (50 min) and 65.61 mg/100 g (control), respectively. Also, the 3,4-dihydroxybenzoic acid quantities of unroasted pumpkin seeds extracted in both extraction systems were determined to be between 11.61 (50 min) and 15.55 mg/100 g (control) and 8.17 (50 min) and 10.77 mg/100 g (25 min), respectively. The catechin quantities of the extracts obtained by water bath and ultrasonic bath extraction of roasted pumpkin seeds were identified to be between 54.83 (50 min) and 86.82 (25 min) and 62.88 (25 min) and 156.86 mg/100 g (control), respectively. Gallic acid contents of roasted pumpkin seeds extracted in both extraction systems were specified between 15.78 (50 min) and 22.54 mg/100 g (control) and 17.61 (50 min) and 21.95 mg/100 g (control), respectively (Table 4). Also, the 3,4-dihydroxybenzoic acid amounts of the extracts obtained by both extraction systems of roasted pumpkin seeds were determined as 9.85 (50 min) and 17.22 mg/100 g (control) to 11.17 (25 min) and 13.74 mg/100 g (50 min), respectively. The highest caffeic acid in roasted pumpkin seeds and routine ultrasonic bath and water bath extraction systems was detected in the control groups. This situation may be due to the extraction power of the solvent used, the agitation speed of the water bath, and the sonication frequency value of the ultrasonic bath. In general, the phenolic compound amounts of roasted pumpkin seeds were higher than those of unroasted ones. This situation may be due to the biochemical reactions that occur due to the heat effect during roasting. Other phenolic constituents (except the predominant phenolic constituents) were found in very low amounts in both unroasted and roasted pumpkin seeds. In addition, this increase sometimes resulted in significant increases at extraction times. This may be due to the agitation speed and the degree of impact of the ultrasonic waves and roasting temperature on the seed cells [19,28]. Ferulic acid amounts of pumpkin seeds were characterized as 5.04 mg/100 g (Çeltik/Konya), 4.97 mg/100 g (Çumra/Konya), 5.17 mg/100 g (İçeri Çumra/Konya) and 4.72 mg/100 g (Polatlı/Ankara) [29]. In the same study, caffeic acid was defined as 3.75 mg/100 g (Çeltik/Konya), 3.68 mg/100 g (Çumra/Konya), 3.83 mg/100 g (İçeri Çumra/Konya) and 3.41 mg/100 g (Polatlı/Ankara) [29]. In another study, unroasted pumpkin seeds contained 60.59 µg/100 g caffeic acid, 36.70 µg/100 g quinine and 0.41 µg/100 g cryogenic acid [27]. The gallic acid content of lump pumpkin seeds was determined to be between 2.13 (microwave) and 8.53 (oven) [28]. The main reason for the differences between present phenolic compounds and results of previous studies may be due to the variety, climatic factors, harvest time, genetic structure and heat treatment norm.

### 3.4. Fatty Acid Compositions of Unroasted and Roasted Pumpkin Seed Oils

Fatty acids of unroasted and roasted pumpkin seed oils extracted with petroleum ether at different times (25 and 50 min) were determined by GC, and their quantitative values are given in Table 5. The applied extraction treatments and times were partially effective on the fatty acid amounts of pumpkin seed oils. The linoleic acid values of unroasted pumpkin seed oils extracted in a water bath and an ultrasonic bath varied between 52.34 (50 min) and 53.33% (control) and 52.90 (50 min) and 53.04% (control), respectively. Linoleic acid amounts of the oils were determined to be between 52.30 (50 min) and 52.84% (25 min) and 52.32 (50 min) and 53.46% (25 min), respectively. Also, the oleic acid amounts of pumpkin seed oils varied between 26.76 (25 min) and 27.23% (50 min) and 26.18 (25 min) and 26.59% (50 min), respectively. The palmitic acid amounts of unroasted pumpkin seed oils extracted by water bath and ultrasonic bath ranged from 11.40 (control) and 12.40% (25 min) to 11.99 (25 min) and 12.29% (50 min), respectively.

Palmitic acid values of the oils extracted by both extraction systems of roasted pumpkin seeds were found to be between 12.40 (25 min) and 12.60% (50 min) and 12.11 (25 min) and 12.15% (control), respectively (Table 6). The stearic acid contents of pumpkin seed oils obtained in both extractions were determined between 7.35% (50 min in water bath) and 7.50% (control in ultrasonic bath). The amounts of other fatty acids were found below 0.48%. Differences in fatty acid amounts may be due to the preservation of the stability and structure of fatty acids during the sonication process. Neđeral et al. [30] detected 49.33% linoleic, 33.90% oleic, 10.54% palmitic and 4.96% stearic acids in roasted pumpkin seed oils. In addition, Topak pumpkin seed oils contained between 42.74% (microwave) and 43.09% (control) linoleic acid, while the oils obtained from Sivri pumpkin seeds contained between 44.78% (oven) and 45.24% (microwave) linoleic acid [28]. These fluctuations in fatty acid contents of pumpkin seed oils according to roasting and sonication types may be due to cultural and sonication conditions.

## 4. Conclusions

The brightness value (L*) of the seeds decreased with roasting. Heat treatment and both extraction systems (water bath and ultrasonic bath) were effective on total phenol, total flavonoid and antioxidant activities (DPPH and ABTS) of pumpkin seeds. Antioxidant activities of the extracts obtained from both extraction systems of unroasted and roasted pumpkin seeds exhibited partial fluctuations depending on the extraction type and duration. It was observed that extraction types and times were effective on the phenolic constituents of pumpkin seeds. The highest caffeic acid was detected in roasted pumpkin seeds, and routine ultrasonic bath and water bath extraction systems were used in the control groups. In general, the phenolic compound amounts of roasted pumpkin seeds were higher than those of unroasted ones. The applied extraction treatments and times were partially effective on the fatty acid contents of pumpkin seed oils. The fatty acid amounts of pumpkin seed oils extracted with an ultrasonic bath were generally slightly higher than those extracted with a water bath.

## Figures and Tables

**Table 1:** Moisture contents and color values of unroasted and roasted pumpkin seeds.

Sample	Moisture Content (%)	*L*	*a*	*b*
Unroasted	8.05 ± 0.72 a *	66.52 ± 1.92 a	−2.14 ± 0.09 a	14.68 ± 0.48 b
Roasted	2.56 ± 0.50 b **	63.38 ± 0.80 b	1.83 ± 0.29 b	25.15 ± 0.77 a

* standard deviation; ** values within each column followed by different letters are significantly different at *p* < 0.05; *n:*3.

**Table 2:** Bioactive properties of unroasted and roasted pumpkin seed extracts obtained by water- and ultrasonic bath systems.

Sample	Process	Time	Total Phenolic Content (mgGAE/100 g)	Total Flavonoid Content (mg/100 g)	Antioxidant Activity (DPPH, mmol/kg)	Antioxidant Activity (ABTS, mmol/kg)
Unroasted	Water bath	Control	7.58 ± 0.14 c *	44.05 ± 2.97 c	0.86 ± 0.00 bc	0.01 ± 0.00 c
		25 min	11.55 ± 0.86 a **	65.48 ± 3.60 a	0.96 ± 0.00 a	0.25 ± 0.00 a
		50 min	7.98 ± 0.24 b	58.33 ± 4.36 b	0.88 ± 0.00 b	0.13 ± 0.00 b
	Ultrasonic bath	Control	7.02 ± 0.00 c	38.33 ± 3.60 c	0.93 ± 0.01 b	0.03 ± 0.00
		25 min	11.71 ± 0.36 b	50.24 ± 2.18 b	0.96 ± 0.00 a	0.29 ± 0.00 a
		50 min	13.93 ± 0.41 a	65.48 ± 4.59 a	0.95 ± 0.00 a	0.28 ± 0.01 abc
Roasted	Water bath	Control	10.20 ± 0.77 c	48.81 ± 0.82 c	0.93 ± 0.00 b	0.01 ± 0.00 c
		25 min	16.55 ± 0.41 b	112.14 ± 2.86 a	1.01 ± 0.01 a	0.35 ± 0.00 a
		50 min	17.18 ± 0.14 a	70.71 ± 3.78 b	1.02 ± 0.00 a	0.28 ± 0.00 b
	Ultrasonic bath	Control	11.47 ± 0.27 c	58.81 ± 0.82 a	0.93 ± 0.01 c	0.10 ± 0.00 c
		25 min	14.96 ± 1.76 b	52.62 ± 3.60 b	0.99 ± 0.00 b	0.22 ± 0.00 b
		50 min	18.69 ± 0.48 a	48.81 ± 2.97 c	1.02 ± 0.00 a	0.40 ± 0.00 a

* Standard deviation; ** values within each column followed by different letters are significantly different at *p* < 0.05; *n*:3.

**Table 3:** Phenolic compounds of unroasted pumpkin seed extracts obtained by water- and ultrasonic bath systems.

Phenolic Compounds (mg/100 g)	Water Bath			Ultrasonic Bath		
	Control	25 min	50 min	Control	25 min	50 min
Gallic acid	28.89 ± 3.59 a *	25.55 ± 0.63 b	23.31 ± 0.05 c	2.31 ± 0.60 c	23.59 ± 1.05 a	7.31 ± 0.11 b
3,4-Dihydroxybenzoic acid	15.55 ± 1.22 a **	12.43 ± 1.31 b	11.61 ± 0.06 c	8.41 ± 1.56 b	10.77 ± 0.43 a	8.17 ± 1.32 c
Catechin	46.95 ± 3.62 b	76.25 ± 1.67 ac	44.50 ± 3.00	65.61 ± 5.48 a	56.21 ± 1.10 b	48.59 ± 4.53 c
Caffeic acid	3.85 ± 1.26 a	2.29 ± 0.55 b	1.36 ± 0.17 c	3.28 ± 0.94 a	2.05 ± 0.28 c	3.26 ± 0.49 ab
Syringic acid	6.22 ± 2.60 a	3.40 ± 0.94 b	1.62 ± 0.28 c	1.36 ± 0.24 b	0.83 ± 0.16 c	1.63 ± 0.34 a
Rutin	1.67 ± 0.37 c	2.28 ± 0.52 ab	2.48 ± 0.46 a	16.44 ± 1.74 a	2.56 ± 0.37 c	9.23 ± 2.11 b
p-Coumaric acid	0.64 ± 0.26 a	0.23 ± 0.08 bc	0.26 ± 0.01 b	0.38 ± 0.08 a	0.13 ± 0.02 c	0.26 ± 0.04 b
Ferulic acid	1.21 ± 0.66 a	0.22 ± 0.01 b	0.19 ± 0.05 c	1.03 ± 0.29 a	0.10 ± 0.02 c	0.36 ± 0.11 b
Resveratrol	0.53 ± 0.02 c	0.62 ± 0.03 b	0.65 ± 0.05 a	0.59 ± 0.03 a	0.53 ± 0.06 b	0.45 ± 0.12 c
Quercetin	1.14 ± 0.11 a	1.05 ± 0.04 b	0.76 ± 0.05 c	0.68 ± 0.12	0.52 ± 0.01	1.28 ± 0.16
Cinnamic acid	0.13 ± 0.02 c	0.16 ± 0.01 b	0.18 ± 0.04 a	0.26 ± 0.01 c	0.32 ± 0.03 b	0.52 ± 0.03 a
Kaempferol	0.46 ± 0.10 b	0.48 ± 0.13 a	0.37 ± 0.10 c	0.28 ± 0.03 b	0.27 ± 0.06 bc	0.35 ± 0.07 a

* Standard deviation; ** values within each row followed by different letters are significantly different at *p* < 0.05; *n*:3.

**Table 4:** Phenolic compounds of roasted pumpkin seed extracts obtained by wate-r and ultrasonic bath systems (mg/100 g).

Phenolic Compounds (mg/100 g)	Water Bath			Ultrasonic Bath		
	Control	25 min	50 min	Control	25 min	50 min
Gallic acid	22.54 ± 2.58 a *	17.17 ± 0.24 b	15.78 ± 0.70 c	21.95 ± 1.15 a	20.08 ± 2.21 b	17.61 ± 1.06 c
3,4-Dihydroxybenzoic acid	17.22 ± 0.82 a **	12.83 ± 1.24 b	9.85 ± 1.29 c	13.26 ± 0.85 ab	11.17 ± 0.34 c	13.74 ± 0.41 a
Catechin	57.71 ± 1.92 b	86.82 ± 3.21 a	54.83 ± 4.48 c	156.86 ± 6.21 a	62.88 ± 2.82 c	135.44 ± 2.31 b
Caffeic acid	5.55 ± 0.82 a	1.39 ± 0.16 b	1.26 ± 0.05 c	5.13 ± 1.99 a	2.92 ± 0.69 c	3.92 ± 0.37 b
Syringic acid	4.71 ± 0.61 a	0.82 ± 0.26 b	0.36 ± 0.05 c	5.59 ± 2.21 b	2.40 ± 0.68 c	7.30 ± 0.47 a
Rutin	21.86 ± 3.99 a	1.93 ± 0.16 c	2.13 ± 0.50 b	5.52 ± 1.97 b	5.22 ± 1.96 c	15.68 ± 0.15 a
p-Coumaric acid	0.85 ± 0.24 a	0.08 ± 0.01 c	0.17 ± 0.04 b	0.44 ± 0.16 a	0.15 ± 0.01 b	0.10 ± 0.00 c
Ferulic acid	1.45 ± 0.59 a	0.14 ± 0.04 c	0.25 ± 0.09 b	1.11 ± 0.52 a	0.06 ± 0.00 c	0.12 ± 0.01 b
Resveratrol	0.52 ± 0.03 aa	0.50 ± 0.10 b	0.40 ± 0.06 c	0.58 ± 0.07 ab	0.59 ± 0.12 a	0.55 ± 0.02 c
Quercetin	1.03 ± 0.09 b	1.24 ± 0.19 a	0.82 ± 0.08 c	1.04 ± 0.27 bc	1.30 ± 0.09 a	1.08 ± 0.13 b
Cinnamic acid	0.19 ± 0.02 c	0.31 ± 0.04 a	0.26 ± 0.00 b	0.41 ± 0.03 a	0.30 ± 0.04 bc	0.32 ± 0.03 b
Kaempferol	0.55 ± 0.15 a	0.26 ± 0.08 c	0.49 ± 0.12 b	0.41 ± 0.10 b	0.45 ± 0.20 a	0.28 ± 0.10 c

* Standard deviation; ** values within each row followed by different letters are significantly different at *p* < 0.05; *n*:3.

**Table 5:** Fatty acid composition of the oils extracted from unroasted pumpkin seeds treated by water- and ultrasonic bath systems.

Fatty Acids	Water Bath			Ultrasonic Bath		
	Control	25 min	50 min	Control	25 min	50 min
Myristic (C14:0)	0.13 ± 0.03 c *	0.23 ± 0.04 b	0.36 ± 0.02 a	0.17 ± 0.00 b	0.29 ± 0.01 a	0.11 ± 0.00 c
Palmitic (C16:0)	11.40 ± 0.04 c **	12.40 ± 0.21 a	11.90 ± 0.24 b	12.17 ± 0.04 ab	11.99 ± 0.01 c	12.29 ± 0.02 a
Stearic (C18:0)	7.43 ± 0.02 a	7.34 ± 0.05 b	7.33 ± 0.05 b	7.28 ± 0.01 b	7.19 ± 0.01 c	7.33 ± 0.01 a
Oleic (C18:1)	26.87 ± 0.03 b	26.76 ± 0.01 c	27.23 ± 0.05 a	26.55 ± 0.02 ab	26.18 ± 0.00 c	26.59 ± 0.01 a
Linoleic (C18:2)	53.33 ± 0.07 a	52.58 ± 0.21 b	52.34 ± 0.12 bc	53.04 ± 0.04 a	52.91 ± 0.00 b	52.90 ± 0.00 b
Arachidic (C20:0)	0.48 ± 0.03 a	0.43 ± 0.00 c	0.45 ± 0.02 b	0.44 ± 0.01 a	0.43 ± 0.02 b	0.43 ± 0.00 b
Linolenic (C18:3)	0.26 ± 0.01 b	0.16 ± 0.00 c	0.28 ± 0.00 a	0.23 ± 0.00 bc	0.91 ± 0.02 a	0.24 ± 0.00 b
Behenic (C22:0)	0.11 ± 0.01 a	0.09 ± 0.00 c	0.10 ± 0.01 b	0.09 ± 0.00 a	0.08 ± 0.00 b	0.07 ± 0.00 c

* standard deviation; ** values within each row followed by different letters are significantly different at *p* < 0.05; *n*:3.

**Table 6:** Fatty acid composition of the oils extracted from roasted pumpkin seeds treated by water- and ultrasonic bath systems (%).

Fatty Acids	Water Bath			Ultrasonic Bath		
	Control	25 min	50 min	Control	25 min	50 min
Myristic (C14:0)	0.28 ± 0.00 a *	0.27 ± 0.01 ab	0.17 ± 0.01 c	0.14 ± 0.01 b	0.10 ± 0.00 c	0.15 ± 0.00 a
Palmitic (C16:0)	12.49 ± 0.07 b **	12.40 ± 0.03 c	12.60 ± 0.25 a	12.15 ± 0.44 a	12.11 ± 0.54 c	12.13 ± 0.06 b
Stearic (C18:0)	7.43 ± 0.02 a	7.44 ± 0.00 a	7.35 ± 0.04 b	7.50 ± 0.09 a	7.42 ± 0.12 bc	7.43 ± 0.04 b
Oleic (C18:1)	26.31 ± 0.01 bc	26.34 ± 0.00 b	26.86 ± 0.06 a	26.29 ± 0.13 b	26.05 ± 0.23 c	27.26 ± 0.07 a
Linoleic (C18:2)	52.68 ± 0.03 b	52.84 ± 0.01 a	52.30 ± 0.17 c	53.10 ± 0.16 b	53.46 ± 0.15 a	52.32 ± 0.11 c
Arachidic (C20:0)	0.44 ± 0.00 b	0.46 ± 0.02 a	0.44 ± 0.01 b	0.47 ± 0.04 b	0.45 ± 0.04 c	0.48 ± 0.02 a
Linolenic (C18:3)	0.28 ± 0.00 a	0.16 ± 0.00 c	0.18 ± 0.00 b	0.25 ± 0.01 b	0.30 ± 0.00 a	0.19 ± 0.01 c
Behenic (C22:0)	0.10 ± 0.00 b	0.11 ± 0.01 a	0.11 ± 0.00 a	0.10 ± 0.02 bc	0.11 ± 0.01 b	0.12 ± 0.00 a

* Standard deviation; ** values within each row followed by different letters are significantly different at *p* < 0.05; *n*:3.

## Data Availability

The original contributions presented in the study are included in the article, further inquiries can be directed to the corresponding author.
